# The role of chronological age in climate change attitudes, feelings, and behavioral intentions: The case of null results

**DOI:** 10.1371/journal.pone.0286901

**Published:** 2023-06-21

**Authors:** Liat Ayalon, Senjooti Roy

**Affiliations:** Louis and Gabi Weisfeld School of Social Work, Bar Ilan University, Ramat Gan, Israel; University of Essex, UNITED KINGDOM

## Abstract

Past research has stressed the role of age and generation in climate change discourse, worries, and willingness to act. Therefore, the present paper aimed to examine the role of chronological age (as an arbitrary factor, which represents ageism) in lay people’s climate change-related attitudes, feelings, and behavioral intentions. Two experiments in different countries, Australia and Israel, were conducted for this purpose. The first study examined the impact of the age of the speaker, who provides information about the climate crisis and the second examined the impact of the age of the group being blamed for the situation. Outcome variables included perceived responsibility and motivation for the current climate situation in study 1 and perceived climate change-related attitudes, feelings, and behavioral intentions in study 2. In study 1 (*n* = 250, Australia), the age of the speaker, a climate activist, varied randomly to test the hypothesis that a younger activist would be more influential and increase motivation and responsibility to act compared to an older activist. In study 2 (*n* = 179, Israel), the age (young vs. old) of the group identified as being responsible for the climate crisis varied randomly, to test the hypothesis that people would be more willing to identify older people as being responsible for the current climate situation, and this would impact climate change-related attitudes, feelings, and behavioral intentions. Both studies resulted in null effects. Additionally, there was no interaction between the age of the respondent and the age of the source of the message or the age group being blamed by the message. The present study has failed to show that strategies that emphasize intergenerational conflict and ageism impact people’s attitudes, feelings, and behavioral intentions towards the current climate situation. This possibly can serve as an instigator for strategies that emphasize intergenerational solidarity, rather than conflict, as a guiding principle in future campaigns that advocate climate change adaptation and mitigation measures.

## Introduction

The climate movement has gained attention in recent years [1,2]. The movement aims to increase awareness about human responsibility toward the changing climate and the importance of identifying sustainable sources of food, energy, and livelihood. Both mitigation and adaptation efforts are currently lagging, and the movement represents an active effort to ensure an urgent and sharp response to current policy shortcomings in handling the climate crisis. These messages and goals are in line with the recent report of the Intergovernmental Panel on Climate Change (IPCC), which has stressed the urgent need for mitigation measures to counter anthropogenic impacts on climate [3].

Age, gender, and geographic location play an important role in the context of climate change. Research exploring the characteristics of climate activists has identified younger girls and women as prominent leaders [4,5]. Climate deniers, on the other hand, largely tend to be older men from the developed world [6,7]. Age, gender, and geographic location also result in differential impacts of climate change. The general claim is that younger people, women, and people from the developing world are more likely to be affected by environmental changes but are less likely to have the political power to influence mitigation and adaptation measures [8–10]. Although, clearly, younger people are impacted by the changing climate with effects expected to intensify and become prolonged over time [11], older people as well are highly susceptible to the negative effects of climate change [12,13].

### The negative impact of the changing climate

The changing climate has had irreversible effects on land, atmosphere, ecosystems, and all living creatures, including humans [3]. It has not only resulted in rise in temperatures, but has also led to more extreme and severe weather changes. Droughts, floods, hurricanes, tornados, the melting of glaciers, rising sea levels, and other extreme weather events have become more frequent and severe because of human (in)action. These extreme changes have affected food production, disease spread, reduction in biodiversity, and damage to ecosystems and infrastructures [3].

It is safe to conclude that the changing climate is impacting our access to clean water, food, and shelter. This has led the UN High Commissioner of Human Rights to conclude that the changing climate poses a real threat to our basic human rights [14]. There is plenty of research to demonstrate the health effects of the changing climate [15,16]. These effects have been documented primarily among young children and older people, leading experts to argue that climate change is one of the leading threats to our health and wellbeing [15,17,18]. Children and young adults are particularly susceptible to the negative effects of the changing climate not only at the present, but also in years to come. Moreover, given the expectation for increasingly warmer climate and more extreme temperature changes in the future, it is expected that the younger generations today will have to adjust to substantially more challenging and extreme climates as they grow older.

Nearly 90% of the burden attributed to disease associated with climate change occurs in children under the age of 5 [19]. Environmental conditions associated with greenhouse gas emission can result in respiratory diseases, sunburn, melanoma, and immunosuppressive diseases. Climate change may also result in drowning, heatstroke, and gastrointestinal diseases. In addition, the indirect effects of the changing climate may result in malnutrition, allergies, vector-borne diseases, and infectious diseases [20]. Other indirect effects may result in loss of livelihood, migration, and exposure to extreme poverty [18]. Depression, post-traumatic stress disorder, and anxiety are some of the mental health consequences among children and youth [18]. This susceptibility among children is attributed to their lifestyle which is characterized by a greater exposure to environmental hazards, as well as to greater sensitivity to environmental exposures due to immature physiological development and dependence on caregivers [21].

Older people as well are considered a high-risk group [15]. This is partially attributed to pre-existing conditions and immune deficiency, but also to a greater reliance on caregivers, poorer social support, limited access to information, inadequate infrastructure and living environment, and poorer financial status [22,23]. Heat, temperature variability, and air pollution are risk factors for mortality due to cardiovascular and respiratory diseases [24,25]. Air pollution is also a risk for dementia [26]. Additionally, older people are susceptible to the negative mental health impact of climate change events, including depression, anxiety, and posttraumatic disorder [9,27]. Nonetheless, compared with older people, younger persons are more likely to report eco-anxiety directly related to fears and concerns associated with the changing climate [28]. This could possibly be attributed to the fact that the younger generations are expected to face more severe climate conditions for a longer period of their lives.

### Climate change, age, and ageism

The present paper aims to examine the role of chronological age in lay people’s attitudes, feelings, and behavioral intentions towards climate change. By focusing on chronological age as an arbitrary determinant of people’s attitudes, feelings, and behavioral intentions, the present study examines the role of ageism in the context of climate change. Ageism is defined as stereotypes, prejudice and discrimination on the basis of age [29]. It can be either positive or negative and can be directed towards any age group [30]. In the context of the present paper, we argue that if people respond differently to messages made by a younger versus an older person or to messages that blame younger versus older people, this differential response represents ageism.

Age and ageism have received increasing attention in relation to climate change in recent years [31–33]. A substantial portion of this body of research has been devoted to intergenerational solidarity and cooperation [31]. Research has stressed varied ways to promote older people as knowledge bearers of traditional methods and skills to preserve the environment [34,35] and younger people as educators, who can teach older people about recycling methods and their significance [36]. At the same time, however, there has been a growing attention to intergenerational tension and conflict, including ageist attitudes and behaviors directed towards both younger and older people [4,32,37].

Several studies have examined public attitudes towards the youth-led climate movement [4,33]. The studies have concluded that the movement is portrayed in a ridiculous light and presented as a whimsical act of youth of limited knowledge, rather than as a real movement which promotes important societal messages and ideology. Older people as well have received their share of criticism regarding their carbon footprint and limited involvement in the climate movement. Specifically, older people have been blamed for not acting on time and for failing to preserve the environment [9,31,37,38]. In addition to being blamed for their substantial carbon footprint, older people have also been viewed as failing to use their political power to influence climate adaptation and mitigation policies. Instead, they have been portrayed as “greedy geezers”, with limited concern and interest in the welfare of future generations by activists and lay people alike [38]. In addition, research has found an association between higher levels of ageism and more worries about climate change as well as greater willingness to act, with those reporting higher levels of ageism toward older generations also being more likely to report greater climate change worries and willingness to act [32].

### The present study

Chronological age has played a substantial role in the climate movement, which is characterized by the young ages of its members [4]. Chronological age also has played a substantial role in much of the discourse generated by the climate movement, which emphasizes both intergenerational solidarity and tension [39]. Nevertheless, the emphasis on chronological age in the context of climate change is somewhat arbitrary, and supposedly affects climate change stereotypes, behaviors, and feelings, thus representing a form of ageism and a source of intergenerational tension. Therefore, the present study aimed to examine the role of age(ism) in the context of climate change. This was done through an experimental design to test the effects of chronological age both in the context of the age of the speaker and in the context of the age of the group being blamed for the current climate situation. To increase the generalizability of the study, we conducted two experiments using two different samples, recruited in two different countries: Australia and Israel. Whereas both countries are affected by the changing climate [40,41], Australians seem to be more vocal about it [42]. To our knowledge, this is the first experimental design to test the impact of chronological age on climate change attitudes, feelings, and behavioral intentions.

The present study reports on two experimental studies that manipulated the age of the source of a climate-related message and the age of the group being blamed for the current climate situation (e.g., the content of the message) to examine their impact on attitudes, feelings, and behavioral intentions towards climate change. Hypotheses were derived from current climate discourse, which tends to emphasize the role of older people as being responsible for the climate crisis and the role of younger people as being involved in the climate movement [5,9,31]. Because past research has found that higher levels of ageism are associated with more climate change worries and greater willingness to act [32], we expected ageism to impact people’s climate change-related attitudes, feelings, and behavioral intentions in these studies as well.

Past research has stressed the role of the speaker (the person who carries the message, i.e., the source) and the contents of the message in shaping people’s attitudes. For instance, source credibility and source-receiver (dis)similarity were identified as important in persuading people to change their attitudes [43,44], with more credible sources being more likely to be persuasive [45]. The content of the message also plays a role, with research identifying “blame-giving” as a persuasive technique under certain circumstances [46,47].

In study 1, we examined the hypothesis that a younger activist would be more influential and increase motivation and responsibility to act compared to an older activist. This hypothesis was derived by the current visibility of the youth-led climate movement [48]. In study 2, we examined the hypothesis that people would be more willing to identify older people as being responsible for the current climate situation and that this would impact their climate change-related attitudes, feelings, and behavioral intentions. This hypothesis was derived from the current discourse which tends to blame older people for their carbon footprint and for their failure to act to prevent the climate crisis [9]. Both hypotheses also were examined in relation to the age of the respondent, with the expectation that, compared with older people, younger people would be more accepting of younger people as the source of the message and more responsive to the blaming of older people.

### Ethics statement

Both studies were approved by the ethics committee of the School of Social Work at Bar Ilan University (# 022103). All participants received written information about the study including confidentiality of participant identity and possible risks and benefits associated with participating in the study. Prior to starting the survey, respondents had to provide informed consent by pressing a button, indicating their agreement to participate in the study. They were allowed to leave the study at any time. Only people over the age of 18 were allowed to participate in the study.

## Study 1

### Materials and methods

#### Sample and procedure

To detect a medium effect size with an alpha of .5 and a power of .8, a sample size of 31 per research arm was deemed adequate. In total, 250 participants from Australia completed an online survey administered via a survey agency. Participants were reimbursed for their time and were allowed to leave the survey at any point. The average age of respondents was 45.5 (SD = 19.6), the majority were women (129; 51.6%) with undergraduate education (95; 38%).

The non-representative sample was recruited via an Australian survey agency. Respondents received a link that directed them to a Qualtrics survey. Respondents received financial compensation for their time. After providing demographic information, respondents were randomly assigned to a manipulation, which consisted of a short vignette which varied by the age of the speaker, as detailed below. They were subsequently asked about their sense of responsibility for the current climate situation and their motivation to combat climate change as the two outcome variables.

#### Manipulation

Respondents were asked to read the following description, with the age of the activist being randomly presented: “According to a **16/40/61/72**-year-old climate activist, “Older people bear much more of the responsibility [for climate change] than the younger generations. For young people, this means turning to those who have responsibility and make the decisions. For the older generation, this means understanding that they are part of the solution.”

#### Outcome variables

Following this statement, respondents were asked how *responsible* they feel for having contributed to climate change and how *motivated* they feel towards combating climate change. Response options range between 1 = not at all and 7 = highly agree. These two items were selected based on a scoping review of existing literature on climate change and intergenerational relations [31] as well as analysis of climate activists’ communication [39].

#### Demographic variables

Age, gender, and education data were gathered based on self-report.

#### Analysis

To calculate whether the age of the speaker climate activist influences sense of responsibility and motivation, Multivariate-Analysis of Variance (MANOVA) was used, with responsibility and motivation as dependent variables, and the age of the speaker activist as the independent variable. Prior to conducting the MANOVA, the MANOVA assumptions were tested [49], including linearity of outcome variables, multivariate normality, and homogeneity of variances and covariances. Next, we examined whether age moderates these associations. SPSS version 26 was used for statistical analysis [50].

### Results

Table 1 presents the distribution of the two outcome variables: responsibility and motivation by the age of the speaker activist. As was previously suggested [51], linearity of the outcome variables was computed via Pearson’s correlations, with a significant moderate correlation (*r* = .54, *p* < .001), confirming linearity. Multivariate normality was assessed by the Cook’s D values. Because the maximum value of the Cook’s D was .037, there was no indication of possible outliers. Multivariate homogeneity of variance and covariance was assessed via the Levene’s test and the Box’s M test, respectively. Levene’s tests are reported in S1 Table. The Levene’s tests were non-significant suggesting that the variances do not significantly differ. Box’s M test was 26.30, *p* = .002. Given Box’s M test’s sensitivity, only p < .001 is considered significant [52], suggesting that all assumptions were met. Table 2 presents the MANOVA results. There were no significant differences in responsibility or motivation by the age of the speaker activist (F(3) = .44, *p* = .73; F(3) = .04, *p* = .99, respectively). This means that the age of the speaker does not impact people’s sense of climate responsibility or motivation to engage in climate action. The age of the respondent was a non-significant moderator of the association between the age of the speaker activist and responsibility or motivation. Hence, the lack of climate activist’s age effect remained even when the age of the respondent varied.

**Table 1:** Sense of responsibility and motivation to act as a function of the age of the climate change speaker activist *(n = 250)*.

	Age of the climate change speaker activist			
	16 (*n* = 63)	40 (*n* = 62)	61 (*n* = 62)	72 (*n* = 62)
Responsibility (1–7)	3.79(1.67)	4.05(1.76)	4.29(2.46)	3.87(1.74)
Motivation (1–7)	4.63(2.63)	4.27(1.56)	4.38(1.74)	4.31(1.76)

1 = not at all; 7 = highly agree.

**Table 2:** Tests of between-subjects effects (n = 250).

	Type III sum of squares	df	Mean square	F	p
Responsibility	3.55	3	1.19	.44	.73
Motivation	.36	3	.12	.04	.99

## Study 2

### Materials and methods

#### Sample and procedure

To detect a medium effect size with an alpha of .5 and a power of .8, a sample size of 75 was deemed adequate. In total, 177 participants completed an online survey administered via an Israeli survey agency. Participants were reimbursed for their time and were allowed to leave the survey at any point. The average age of respondents was 52.2 (SD = 21.3), the majority were men (111; 53.1%) with high school education (75; 41.9%).

An Israeli survey company collected the data by sending a Qualtrics link to a non-representative sample. In addition to demographic information, respondents were randomly assigned to one of two vignettes that varied based on the age of the group being blamed for the current climate situation. Subsequently, the outcome variables: beliefs, feelings and behavioral intentions were assessed.

#### Manipulation

Respondents were randomly told that, "many climate change activists claim that **older/younger** people are responsible for the current climate change crisis."

#### Outcome variables

Following the manipulation, respondents were asked to indicate how much they agree or disagree with each of the following statements: a) climate change is real, b) I worry about the effects of climate change on my life, c) I am active in the climate change movement, d) I worry about the effects of climate change on younger people, e) I worry about the effects of climate change on older people. Response options ranged between 1 = not at all and 10 = fully agree. These items were selected following a review of existing measures of climate change as well as analysis of climate activists’ communication [39]. Cronbach’s alpha of the five items was .778.

#### Demographic variables

Age, gender, and education data were gathered based on self-report.

#### Analysis

To calculate differences between respondents exposed to claims concerning the responsibility of older/younger people towards the current climate situation and attitudes, feelings, and behavioral intentions about climate change, a MANOVA was used. Prior to conducting the MANOVA, all relevant assumptions were tested [49]. Next, interactions between the age of the respondent and the age group identified as being responsible for the climate situation were calculated. SPSS version 26 was used for statistical analysis [50].

### Results

Table 3 presents the distribution of attitudes towards climate change as a function of the age of the group being blamed. S2 Table presents the correlations across the dependent variables as an indicator of linearity. There were significant moderate correlations for all variables, except for the belief that climate change is real and willingness to act. Therefore, in an additional sensitivity analysis, these two items were analyzed using two separate One-Way Analysis of Variance (ANOVAs). Results were similar and are available upon request. Multivariate normality was assessed by saving the Cook’s D values. Because the maximum value of the Cook’s D was .048, there was no indication of possible outliers. Multivariate homogeneity of variance and covariance were assessed via the Levene’s test and Box’s M tests, respectively. Levene’s tests are reported in S3 Table. The tests were non-significant suggesting that the variances do not significantly differ. Box’s M test was 9.46, *p* = .87, suggesting that multivariance homogeneity of covariance was met. Table 4 presents the MANOVA results. There were no significant differences on any of the attitudinal items between those exposed to a message that blamed older people vs. those exposed to a message blaming younger people for the current climate change situation (F(1) = .17, p = .66; F(1) = .02, *p* = .90; F(1) = .03, *p* = .87; F(1) = .06, *p* = .80; F(1) = .00, *p* = .99, items a-e respectively). There also were no interactions between the age of the respondent and the age group identified as being responsible for the current climate situation.

**Table 3:** Attitudes towards climate change as a function of the age of the group being blamed for the current climate change situation (n = 179).

	Younger people to blame (n = 91)	Older people to blame (n = 88)
Climate change is real (1–10)	7.08(2.98)	6.90(2.62)
I worry about the effects of climate change on my life (1–10)	5.55(2.61)	5.60(2.72)
I am active in the climate change movement (1–10)	2.93(2.51)	2.87(2.43)
I worry about the effects of climate change on older people in my family (1–10)	4.89(2.87)	4.68(2.90)
I worry about the effects of climate change on younger people in my family (1–10)	5.58(3.11)	5.59(2.87)

1 = not at all; 10 = completely agree.

**Table 4:** Tests of between-subjects effects (n = 250).

	Type III sum of squares	df	Mean square	F	p
Climate change is real	1.48	1	1.43	.19	.67
I worry about the effects of climate change on my life	.12	1	.13	.02	.90
I am active in the climate change movement	.16	1	.16	.03	.87
I worry about the effects of climate change on older people in my family	.54	1	.59	.06	.80
I worry about the effects of climate change on younger people in my family	.00	1	.00	.00	.99

## Discussion

Relying on two experimental designs, this paper examined the role of chronological age (as a proxy of ageism, given its arbitrary nature) in the context of climate change-related attitudes, feelings, and behavioral intentions. In the first experiment, we manipulated the age of the speaker activist, under the assumption that compared with an older speaker, a younger speaker who delivers a message about the current climate situation, would make respondents feel more responsible for and motivated to act towards changing the current climate situation. In the second experiment, the age of the group being blamed for the current climate situation was manipulated, with the expectation that blaming older people would result in increased worries and willingness to act. These hypotheses were inspired by the current climate movement and associated discourse, which is largely being led by younger activists and tends to blame older people for their inaction [5,9,31]. Both hypotheses were rejected and there were no interactions between the age of the activist speaker and the age of the respondent nor between the age of the group being blamed and the age of the respondent.

Our findings demonstrate that the age of the speaker activist and the age of the group being blamed for the current climate situation have no impact on people’s attitudes. This stands in contrast to past research which has emphasized intergenerational conflict and ageism in climate change discourse [4,31]. It is important to note that the present findings do not preclude the presence of intergenerational conflict or ageism in the context of climate change. However, they do suggest that the chronological age of the person carrying the message and the age of the group identified as being responsible for the current climate situation are of limited impact on people’s climate change attitudes, feelings, and behavioral intentions. Moreover, the null effects remained also when the age of the respondent was examined as a possible moderator. The fact that we relied on experimental designs and two different samples recruited in two different countries further strengthens the reliability and generalizability of our findings.

When reviewing the findings, the present study’s limitations should be acknowledged. First, the sample is non-representative, and we have no documentation of refusals. Second, given the online administration mode, our sample is technologically literate, and it is possible that people with lesser command of online technology hold different attitudes and are influenced differently by sources and messages. In addition, this study relied on a single exposure to varied sources and messages rather than on repeated exposure, which occurs in real life. In addition, only the first and last anchors (e.g., end-labeling) were provided when assessing the outcome variables. This likely has affected the response style. Past research has found that end-labeling results in more extreme responses than full-labeling of all response options [53]. Nevertheless, the present findings are reassuring and possibly have important implications for policy stakeholders. As much of the discourse around climate change and the climate movement has evolved around the age of the activists and the age of the group/s being blamed [5,9,31], the present study suggests that these strategies have limited impact. Whereas strategies that emphasize intergenerational conflict and ageism have limited effect on climate change attitudes, feelings, and behavioral intentions, it is possible that messages that foster intergenerational solidarity and cooperation will have a stronger impact. This possibly can serve as a guiding principle in future campaigns that advocate for climate change adaptation and mitigation measures.

## Conclusions

The present study has failed to identify age(ism) effects in the context of climate change. Although this is reassuring, past research has stressed the role of ageism in the context of climate change worries [32] as well as climate change discourse [4,38]. Our findings suggest that not only are strategies that emphasize the age of the speaker, or the age of the group being blamed for climate change ageist, but they are also ineffective. Therefore, it is important to use the findings of the present study to shape future climate change discourse. If indeed, the age of the speaker and the age of the group being blamed for the current climate situation play little role in people’s climate change attitudes, feelings, and behavioral intentions, then it is important to educate activists about this especially because activists tend to emphasize age and generation in their discourse [31]. Further education about ageism and its negative effects could be beneficial. Past research has stressed the benefits that older people gain from participating in the climate movement [54,55]. Hence, it is important to encourage older people’ participation in the climate movement, while breaking the age barriers that tend to characterize this movement. Future research will benefit from addressing ageism in the context of climate change. This can be done via educational interventions and intergenerational contact in the context of the climate movement. Emphasizing intergenerational solidarity and commitment could be an effective way to bring generations together towards addressing a common societal goal [39].

## Supporting information

S1 Table Levene’s test of equality of error variances. (DOCX)

S2 Table Levene’s test of equality of error variances. (DOCX)

S3 Table Correlations among study variables. (DOCX)
