# Supplementary material for: The role of chronological age in climate change attitudes, feelings, and behavioral intentions: The case of null results
Source: PLoS One. 2023 Jun 21;18(6):e0286901. doi: 10.1371/journal.pone.0286901 (PMC10284386; doi:10.1371/journal.pone.0286901)
Supplement: S1 Table — (DOCX) [file pone.0286901.s001.docx]

**Table S1. Levene’s test of equality of error variances**

| Variable | Statistics | df2(df1) | p-value |
| --- | --- | --- | --- |
| Contribute | 2.01 | 245(3) | .71 |
| Motivate | .72 | 246(3) | .51 |
