# Supplementary material for: The role of chronological age in climate change attitudes, feelings, and behavioral intentions: The case of null results
Source: PLoS One. 2023 Jun 21;18(6):e0286901. doi: 10.1371/journal.pone.0286901 (PMC10284386; doi:10.1371/journal.pone.0286901)
Supplement: S2 Table — (DOCX) [file pone.0286901.s002.docx]

**Table S2. Levene’s test of equality of error variances**

| Variable | Statistics | Df2(df1) | p-value |
| --- | --- | --- | --- |
| Climate change is real | .77 | 177(1) | .38 |
| I worry about the effects of climate change on my life | .26 | 177(1) | .38 |
| I am active in the climate change movement | 1.13 | 177(1) | .29 |
| I worry about the effects of climate change on older people in my family | .02 | 177(1) | .90 |
| I worry about the effects of climate change on younger people in my family | 1.28 | 177(1) | .26 |
