# Supplementary material for: The role of chronological age in climate change attitudes, feelings, and behavioral intentions: The case of null results
Source: PLoS One. 2023 Jun 21;18(6):e0286901. doi: 10.1371/journal.pone.0286901 (PMC10284386; doi:10.1371/journal.pone.0286901)
Supplement: S3 Table — (DOCX) [file pone.0286901.s003.docx]

**Table S3. Correlations among study variables.**

|  | 1 | 2 | 3 | 4 |
| --- | --- | --- | --- | --- |
| 1. Climate change is real (1-10) |  |  |  |  |
| 2. I worry about the effects of climate change on my life (1-10) | .37** |  |  |  |
| 3. I am active in the climate change movement (1-10) | -.00 | .33 |  |  |
| 4. I worry about the effects of climate change on older people in my family (1-10) | .24** | .73** | .51** |  |
| 5. I worry about the effects of climate change on younger people in my family (1-10) | .25** | .69** | .30** | .65** |

**p<.01
